# Optimizing responsiveness to feedback about antibiotic prescribing in primary care: protocol for two interrelated randomized implementation trials with embedded process evaluations

**DOI:** 10.1186/s13012-022-01194-8

**Published:** 2022-02-14

**Authors:** Jennifer Shuldiner, Kevin L. Schwartz, Bradley J. Langford, Noah M. Ivers, On behalf of the Ontario Healthcare Implementation Laboratory study team, Monica Taljaard, Jeremy M. Grimshaw, Meagan Lacroix, Mina Tadrous, Valerie Leung, Kevin Brown, Andrew M. Morris, Gary Garber, Justin Presseau, Kednapa Thavorn, Jerome A. Leis, Holly O. Witteman, Jamie Brehaut, Nick Daneman, Michael Silverman, Michelle Greiver, Tara Gomes, Michael R. Kidd, Jillian J. Francis, Merrick Zwarenstein, Jonathan Lam, Cara Mulhall, Sharon Gushue, Sukhleen Uppal, Andrew Wong

**Affiliations:** 1 Women’s College Hospital, 76 Grenville St, Toronto, ON M5S 1B2 Canada; 2 Dalla Lana School of Public Health – University of Toronto, ICES, Unity Health Toronto, Public Health Ontario, 480 University Ave, Toronto, ON M5G 1V2 Canada; 3 Public Health Ontario, 480 University Ave, Toronto, ON M5G 1V2 Canada; 4 University of Toronto, Women’s College Hospital, 76 Grenville St, Toronto, ON M5S 1B2 Canada

**Keywords:** Audit and feedback, Antibiotic prescribing, Antimicrobial resistance, Process evaluation, Protocol

## Abstract

**Background:**

Audit and feedback (A&F) that shows how health professionals compare to those of their peers, can be an effective intervention to reduce unnecessary antibiotic prescribing among family physicians. However, the most impactful design approach to A&F to achieve this aim is uncertain. We will test three design modifications of antibiotic A&F that could be readily scaled and sustained if shown to be effective: (1) inclusion of case-mix-adjusted peer comparator versus a crude comparator, (2) emphasizing harms, rather than lack of benefits, and (3) providing a viral prescription pad.

**Methods:**

We will conduct two interrelated pragmatic randomized trials in January 2021. One trial will include family physicians in Ontario who have signed up to receive their *MyPractice*: Primary Care report from Ontario Health (“OH Trial”). These physicians will be cluster-randomized by practice, 1:1 to intervention or control. The intervention group will also receive a Viral Prescription Pad mailed to their office as well as added emphasis in their report on use of the pad. Ontario family physicians who have *not* signed up to receive their *MyPractice*: Primary Care report will be included in the other trial administered by Public Health Ontario (“PHO Trial”). These physicians will be allocated 4:1 to intervention or control. The intervention group will be further randomized by two factors: case-mix adjusted versus unadjusted comparator and emphasis or not on harms of antibiotics. Physicians in the intervention arm of this trial will receive one of four versions of a personalized antibiotic A&F letter from PHO. For both trials, the primary outcome is the antibiotic prescribing rate per 1000 patient visits, measured at 6 months post-randomization, the primary analysis will use Poisson regression and we will follow the intention to treat principle. A mixed-methods process evaluation will use surveys and interviews with family physicians to explore potential mechanisms underlying the observed effects, exploring targeted constructs including intention, self-efficacy, outcome expectancies, descriptive norms, and goal prioritization.

**Discussion:**

This protocol describes the rationale and methodology of two interrelated pragmatic trials testing variations of theory-informed components of an audit and feedback intervention to determine how to optimize A&F interventions for antibiotic prescribing in primary care.

**Trial registration:**

NCT04594200, NCT05044052. CIHR Grant ID: 398514

**Supplementary Information:**

The online version contains supplementary material available at 10.1186/s13012-022-01194-8.

Contributions to the literature

- Audit and feedback can act as an effective intervention to reduce unnecessary antibiotic use in primary care.
- This study leverages a pragmatic trial design and a theoretical informed process evaluation using mixed methods to enhance our understanding of antibiotic audit and feedback.
- This large-scale evaluation will evaluate three design modifications of antibiotic A&F that could be readily scaled and sustained if shown to be effective.

## Background

Antibiotic overuse is common and contributes to rising rates of antimicrobial resistance. Primary care physicians prescribe 64% of antibiotics, rendering this group of prescribers as crucial partners in antimicrobial stewardship efforts [1–3]. Audit and feedback (A&F) can act as an effective intervention to reduce unnecessary antibiotic use in primary care [4–7]. More specifically, peer comparison A&F that shows how health professionals compare to those of their peers, can be an effective intervention for reducing antibiotic prescribing volume among family physicians [4, 6, 8]. For A&F to work, recipients must feel that the data showing a gap between their existing and desired practice is both valid and actionable. However, research is needed to examine ways to optimize the effects of A&F by enabling this process [9]. Questions remain regarding the optimal way to incorporate these elements in A&F interventions and there is a need to test different designs comparatively (head-to-head). Furthermore, there is uncertainty about the effects of antibiotic prescribing feedback in the Coronavirus disease 2019 (COVID-19) pandemic context, in which patterns of care (i.e. the increase in virtual care) have meaningfully changed. Analyses have shown that prescribing behaviour has fluctuated since the pandemic [10–13] and this may lead recipients of feedback to dismiss the validity of the audit when they see their prescribing has dropped.

Our Implementation Science Laboratory works with government agencies that conduct A&F to embed research studies within ongoing provincial activities [14] and with these questions in mind, we designed two interrelated trials to evaluate different elements of antibiotic A&F. Please refer to Additional File 1 for a full CONSORT checklist.

### Ontario Health (OH) Trial

In Ontario, A&F is routinely offered to primary care providers from a variety of sources. For example, Ontario Health (OH)—an agency created by the Government of Ontario with a mandate to connect and coordinate the province’s health care system to help ensure that Ontarians receive the best possible care—provides A&F to physicians who voluntarily sign up for their “*MyPractice*: Primary Care” reports and approximately 4750 (of 9500 eligible) Ontario family physicians are signed up to receive these reports. These are multi-topic reports with aggregated (physician-level) data collated from ICES (formerly, the Institute for Clinical Evaluative Sciences; a publicly funded organization which holds administrative health services records for the province of Ontario) and sent twice a year via email to physicians. As of December 2021, the *MyPractice*: Primary Care reports will include data on antibiotic prescribing, along with quality improvement resources such as educational material from Choosing Wisely Canada (a national initiative that seeks to reduce unnecessary tests and treatments in health care), including a link to a viral prescription pad (described below). This initiative provides us the opportunity to test elements of the viral prescription pad in the context of a multi-topic report.

### Public Health Ontario (PHO) Trial

We have also planned a trial with Public Health Ontario (PHO)—an agency of the provincial government responsible for providing scientific and technical advice on matters of public health concern— with primary care physicians that did not sign up for the MyPractice report mentioned above. In Fall 2018, we conducted an A&F trial that tested mailed letters to 3500 family physicians in Ontario, Canada, who prescribe the highest volume of antibiotics. The trial resulted in reduced antibiotic volume and antibiotic prescription durations [15]. This study was conducted with IQVIA data, which is population-based, but could not be adjusted for patient volume or case-mix. During the project, we learned that family physicians questioned the credibility of the report in terms of its ability to fairly account for their practice size and population.

To address these concerns in future A&F interventions in Ontario, it would be necessary to link antibiotic prescribing data to other datasets. Therefore, we were required to use data held at ICES, which is a custodian to a data repository with patient- and physician-level, coded and linkable health data sets in Ontario, Canada. It encompasses publicly funded administrative health services records for the Ontario population eligible for universal health coverage (≈ 98.5%). ICES can therefore link prescriber characteristics, including patient volume, and patient characteristics, including comorbidities, to prescription data. However, prescribing data are complete only for patients 65 years of age and older. While prior work has shown that prescribing for those aged 65+ correlates highly with prescribing for all ages in primary care (see Additional File 2, Table S1, Figure S1-2), it is unclear if the credibility gained by accounting for case-mix will be lost by focusing only on prescribing for elders.

Herein we describe a protocol for two interrelated trials with embedded process evaluations. This large-scale evaluation provides an opportunity to evaluate not only whether A&F is helpful in the COVID-19 pandemic context, but also, how best to design A&F, and to explore why we observed (or not) changes in antibiotic prescribing.

### Research questions

#### Effectiveness questions

##### OH Trial

What is the effect of antibiotic A&F coupled (or not) with a “viral prescription pad” on physician antibiotic prescribing after 6 months?

##### PHO Trial

Do patients of family physicians receiving antibiotic A&F receive and fill fewer antibiotic prescriptions compared to patients of family physicians who do not receive A&F at 6 months after intervention roll out?

(2a) What is the effect of peer comparators adjusted for case-mix on physician antibiotic prescribing after 6 months?

(2b) What is the effect of antibiotic-associated harms on physician antibiotic prescribing after 6 months?

##### Mechanism questions

Did the interventions work better for some physicians or patients than others?

What are the effects of the intervention components on the theory-based constructs hypothesized to explain behaviour change resulting from the intervention?

Why might the intervention have or have not worked in reducing unnecessary antibiotic prescriptions?

## Methods

### General study settings

Ontario, Canada’s most populous province, has a publicly funded healthcare system as mandated by the Canada Health Act, with universal coverage for all medically necessary procedures and visits provided by physicians [16]. The study population includes all primary care (family medicine and general practice) physicians in Ontario in active practice. A description of the PICOT of both trials can be found in Table 1.

**Table 1:** PICOT Table of the Ontario Health Trial and the Public Health Ontario Trial

	OH Trial	PHO Trial
Population	Primary care physicians fully registered to receive the *MyPractice*: Primary Care report	Primary care physicians who have not enrolled to receive the *MyPractice*: Primary Care report
Intervention	A mailed viral prescription pad and emphasis of the viral prescription pad embedded within a multi-topic audit and feedback report	Antibiotic peer comparison audit and feedback report with adjusted comparators and/or harms focused messaging in a 2 × 2 factorial experiment
Comparison	*My practice* report without a mailed viral prescription pad (randomized 1:1)	No adjusted comparators and/or no harms messaging in a 2 × 2 factorial experiment, or no letter (randomized 4:1 letter:control)
Outcome	Antibiotic prescribing rate (APR) defined as the total number of antibiotic prescriptions per 1000 patient visits 65 years of age or older	Antibiotic prescribing rate (APR) defined as the total number of antibiotic prescriptions per 1000 patient visits 65 years of age or older
Time	6 months	6 months

### Trial design

**Fig. 1:**
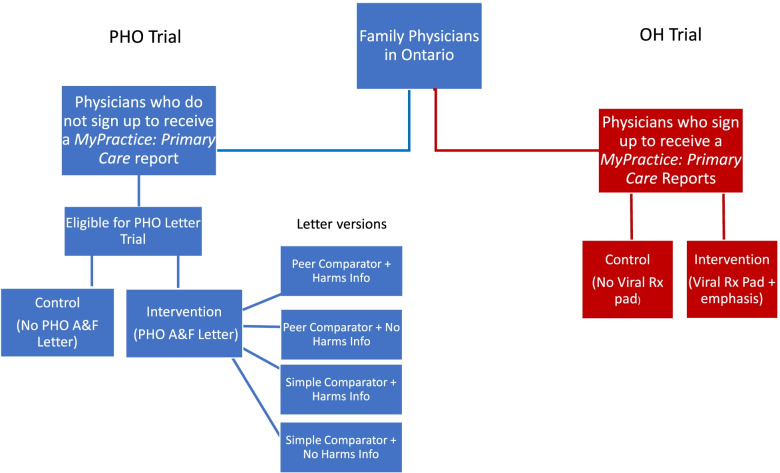
Study design of two linked trials

We will conduct two interrelated, pragmatic trials [17] (Additional File 3 and Table S3) to test different ways to optimize the effects of A&F to reduce antibiotic overprescribing (Fig. 1).

#### OH Trial

Physicians receiving the antibiotic prescribing feedback within the *MyPractice*: Primary Care feedback report will be randomized (1:1) to control group or intervention (physician will receive a viral prescription pad in the mail plus emphasis of the pad in the report).

#### PHO Trial

Eligible physicians will be randomized 4:1 to intervention (an exploratory 2 × 2 factorial experiment for testing and refining interventions [18]) or control. The intervention group will be further randomized by two factors: case-mix adjusted versus unadjusted comparator and emphasis or not on harms of antibiotics. Such designs are especially useful options in health behaviour research to efficiently compare multiple intervention design options, particularly when there is no expectation that the factors being tested will interact meaningfully. We chose to include a control arm in the PHO Trial as there is uncertainty about the effects of antibiotic prescribing feedback in the post-covid context, in which prescribing behaviour has fluctuated [10, 11] and patterns of care (i.e. the increase in virtual care) have meaningfully changed.

### Population and recruitment

#### OH Trial

Family physicians who are fully registered (i.e. whose accounts are activated) to receive the *MyPractice*: Primary Care reports by September 2021 and have sufficient data to populate the report are eligible.

#### PHO Trial

Family physicians who did not sign up by September 2021 to receive the *MyPractice:* Primary Care report are eligible. Physicians who have previously opted out of antibiotic prescribing letters from PHO will also be excluded (*N* ≈ 15). The list of eligible participants will be identified using linked health administrative data at ICES. All participants will receive a notification in the mail 3 weeks prior to randomization, offering them the opportunity to opt out of the trial.

For both trials, we will exclude family physicians with < 100 unique patient visits in the most recent year or in two of the three prior years and/or < 10 antibiotic prescriptions to patients 65+ in the most recent year or two of the three prior years. Such physicians would not have sufficient data to provide meaningful feedback.

### Intervention

#### OH Trial

**Fig. 2:**
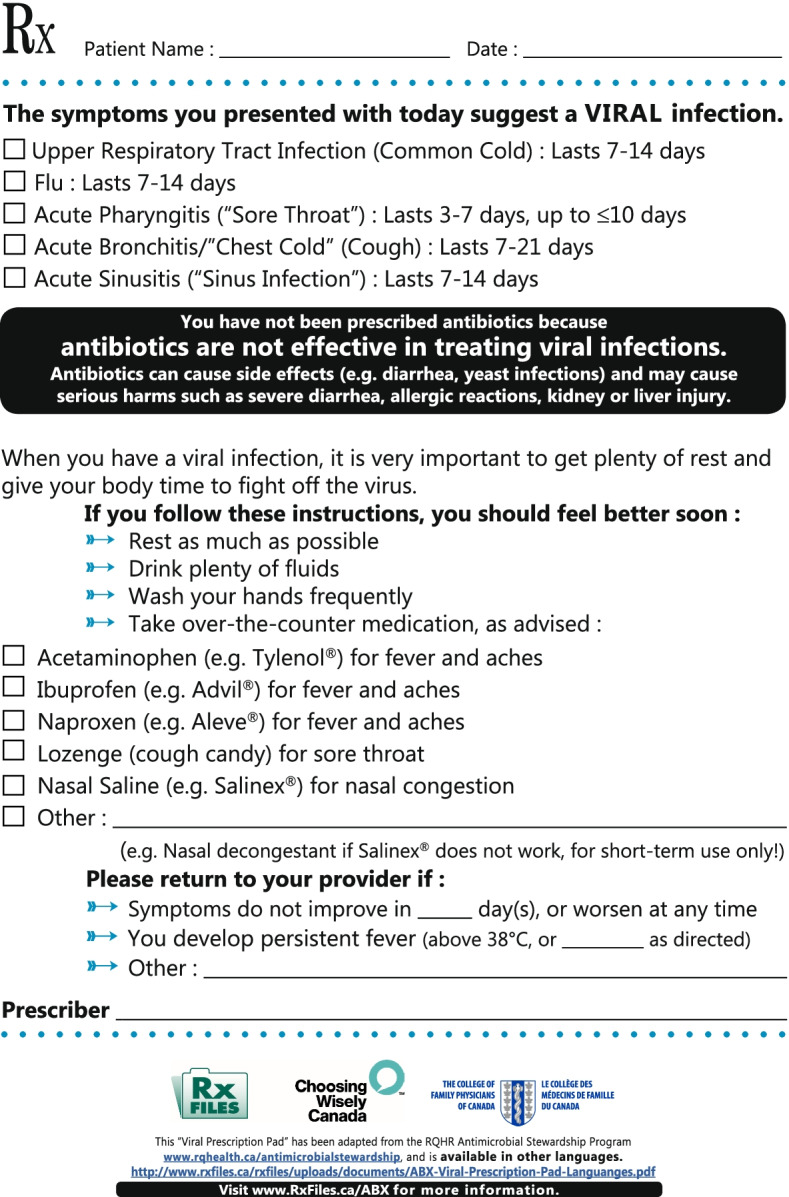
Viral prescription pad

For the OH Trial, physicians who are registered to receive the *MyPractice*: Primary Care report will receive antibiotic prescribing metrics and quality indicators on diabetes, cancer screening, and opioid prescribing. The *MyPractice*: Primary Care report uses a simple comparator, does not emphasize harms, and is sent twice a year. A sample of the *MyPractice*: Primary Care report is available at https://www.hqontario.ca/DesktopModules/Services/API/PhysicianReports/GetSampleReport/My%20Practice%20Primary%20Care%20Physician%20Sample%20Report.pdf. There will also be a link to an electronic version of the Choosing Wisely Canada Viral Prescription Pad (see Fig. 2 below).

##### Intervention group

Mailed Viral Prescription Pad and Emphasis on its Use in the MyPractice report

**Fig. 3:**
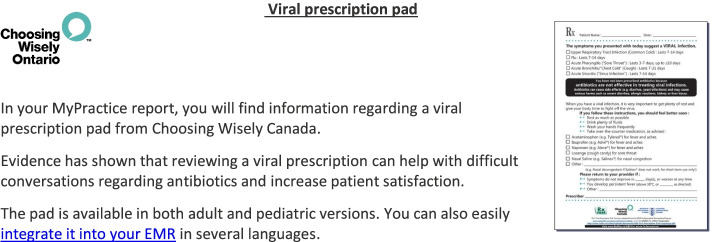
Emphasis on viral prescription pad inserted in *MyPractice*: Primary Care report dissemination email

The usual approach to providing prescribing feedback is to provide quality improvement resources with educational information. The reports of the intervention group will include content to emphasize and encourage use of the viral prescription pad. We will emphasize the viral prescription pad by (1) encouraging it in the dissemination email of the report (see Fig. 3), and (2) mailing a paper version of the viral prescription pad (see Additional File 4). We hypothesize that adding a paper version of this resource and emphasis in the dissemination email will increase self-efficacy in conversations regarding antibiotics and change antibiotic prescribing behaviour.

#### PHO Trial

All intervention recipients except the control group will receive two antibiotic prescribing A&F reports, one in January 2022 and the same report in February 2022, in an attempt to increase salience. The reports will include personalized data regarding total antibiotic dispensing per 1000 patient visits in patients ≥ 65 years of age and proportion of antibiotic prescriptions dispensed for a duration of > 7 days. Physicians prescribing above the 25th percentile will receive their audit and feedback data with this statement: “XX% of your peers with patient and practice characteristics very similar to yours prescribed fewer antibiotics than you did” and a graph with prescribing rates and their comparators (25th and 50th percentiles, Fig. 2). The average primary care physician in Ontario can safely reduce antibiotic use by 25%. Therefore, we used the 25th percentile as an achievable target [19].

The feedback report will be mailed to each physician’s primary practice address. The report will include the prescribing quality indicators, as well as accompanying content informed by a user-centred design process (see Additional File 5). The report will include educational materials from Choosing Wisely Canada, including a viral prescription pad (further described below) and link to the “Cold Standard” Guidance (https://choosingwiselycanada.org/download/5832/).

##### Factor 1

Case-mix adjusted comparator vs unadjusted comparator

**Fig. 4:**
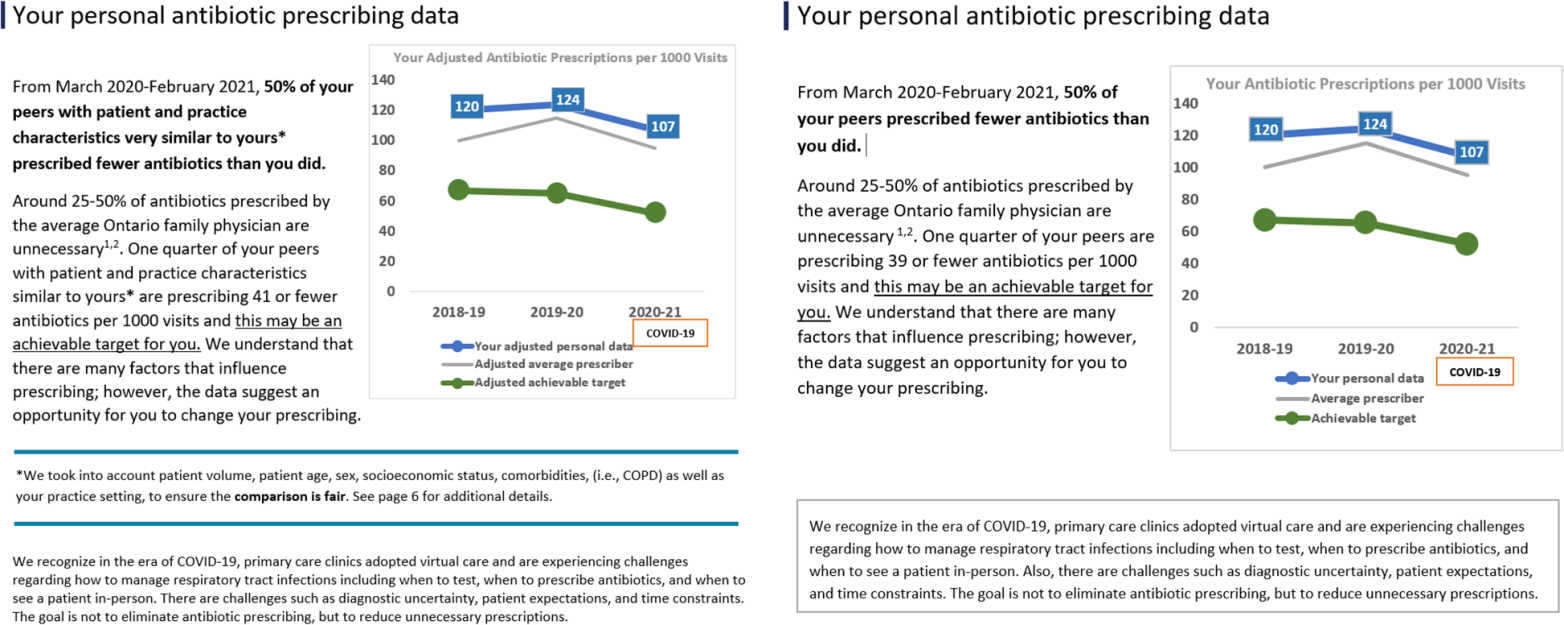
Case-mix adjusted comparator and unadjusted comparator

We included a case-mix adjusted comparator because in our previous study [15] physicians questioned whether it was fair to compare them to others with a different case-mix and context. Recipients’ prescribing rates and their comparators (25th and 50th percentiles) will be adjusted using hierarchical regression modelling incorporating patient volume, patient age, sex, socioeconomic status, and patient comorbidities. We will also adjust for emergency room visits. We hypothesize that comparing to like-peers will change outcome expectancies and intention and thus change antibiotic prescribing behaviour (Fig. 4).

##### Factor 2

Emphasis on risk of harms of antibiotic vs no benefit only

**Fig. 5:**
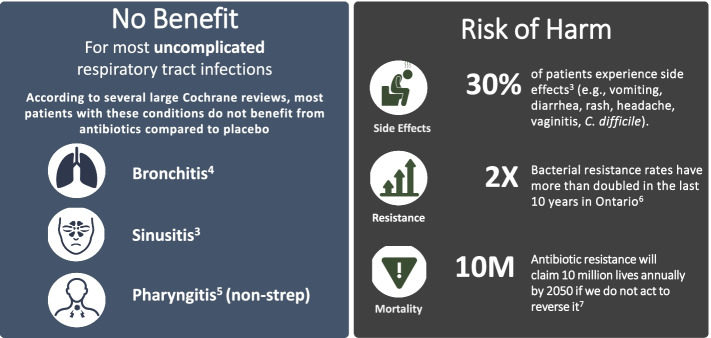
Infographic to be included in the emphases on risk harms of antibiotic group

The usual approach to feedback on antibiotic prescribing tends to focus on lack of benefit. Factor 2 will include an emphasis on potential harms caused by unnecessary use of antibiotics. We will provide an infographic to highlight the frequency of antibiotic side effects and also emphasize the principle of “do no harm” (see Fig. 5). We hypothesize that adding an emphasis on harms will change risk perception and intention and thus change antibiotic prescribing behaviour.

### Allocation

#### PHO Trial

Physicians will be given the opportunity to opt out of the trial. An epidemiologist, not involved in the study, will generate the allocation sequence. Randomization will be conducted at level of the physician, stratified by involvement in our prior PHO letter trial [15]. Like a recent factorial trial testing A&F with a control arm [20], we will allocate physicians 4:1 to intervention or control. Then, we will randomize physicians within the intervention arm to each of the 2 trial factors described above. The randomized lists will then be sent to another independent individual at PHO responsible for intervention implementation. Although physicians commonly work in groups, it is not common practice in Ontario for physicians to share or discuss clinical performance data [21] and we therefore judge the risk of contamination in this trial to be low.

#### OH Trial

We cluster-randomized in this trial since the viral prescription pad may be incorporated into the electronic medical record that is typically shared in an office or practice group. An analyst not involved in the study will stratify by (i) number of physicians consenting to receive their *MyPractice*: Primary Care report (median); (ii) number of patients per consenting physician (volume); (iii) whether the physician belongs to a family health team. This randomization sequence (1:1) will be sent to a member of the OH team who will assign the arms of as either intervention or control, so that the trial analyst will be blinded.

### Outcomes

For both trials, the primary outcome is the antibiotic prescribing rate (APR) defined as the total number of antibiotic prescriptions per 1000 patient visits in those aged 65 or greater from January 1, 2021, to June 30, 2022. Secondary outcomes will be measured cumulatively from January 1 to December 31 2022, at 6 and 12 months, for patients aged 65 or greater, and are as follows: number of antibiotic prescriptions > 7days per 100 total antibiotic prescriptions; number of broad-spectrum antibiotics per 100 total antibiotic prescriptions as defined by Steinman et al. [22]; antibiotic days of therapy (DOT) per 1000 patient visits; antibiotic drug costs; and number of antibiotic prescriptions per 100 65+ patient visits for presumed viral condition (and thus likely unnecessary), using definitions from Schwartz [1] and Silverman [23] (see Additional File 6 and Table S4-6). All secondary outcomes with be measured at 6 and 12 months and APR only at 12 months. The process evaluation will be discussed below.

### Data collection and transfer

For both trials, the data to measure prescribing quality indicators in the feedback report and the outcomes will be derived from the ICES data repository. The province provides publicly funded coverage, with no co-payment, for most medications including commonly used antibiotics through the Ontario Drug Benefit (ODB) plan. The ODB database covers selected populations, including persons aged 65 years or older and has > 99% accuracy [24–26]. Additional ICES databases will be linked and utilized for diagnostic codes, patient demographics, patient comorbidities, and physician characteristics. An ICES analyst will create physician-specific data reports to export to an Excel file with re-identified physician information including name and mailing address.

#### PHO Trial

OH will provide ICES with a list of physicians who have opted-in to the *MyPractice*: Primary Care report. ICES will then generate a physician list for the trial to PHO (excluding those with receive a *MyPractice*: Primary Care report) who will link this list to physician mailing addresses provided by the College of Physicians and Surgeons of Ontario. PHO will send opt-out letters to these physicians. After removing all physicians who opt out, PHO will apply the randomization sequence and then generate and send feedback letters.

#### OH Trial

A randomization sequence will be sent to OH. After it is applied, OH will apply the sequence and then send out two different emails to physicians—one emphasizing the viral prescription pad and one that does not. The viral prescription pad will be mailed to intervention participants with a cover letter describing how, when, and why it should be used.

### Analysis

Our primary analysis was conducted using an intent-to-treat approach [27] and therefore included all randomized physicians. Analysts will be unaware of which group the physician belonged to. We will exclude outliers at the 99th percentile at baseline from each arm to eliminate the implausibly high numbers of antibiotic prescriptions attributed to a small number of physicians [4].

#### OH Trial

The primary analysis will follow the intention to treat principle and use Poisson regression (Table 2). The unit of analysis will be the physician. The model will compare the intervention group (*MyPractice*: Primary Care report plus the mailed viral prescription pad/emphasis) against control (*MyPractice*: Primary Care report only).

**Table 2:** Poisson models measuring primary outcomes over the 6-month post-randomization period

Dependent variable	Offset (denominator)	Adjustment	Additional details
Number of antibiotics	Log of patient visits in those aged 65 or greater	Log of the baseline prescription rate (1 year pre-intervention) as well as physician years since medical school and sex [23]	Robust standard errors will be used, accounting for the correlation of multiple physicians at the same office-location using an exchangeable correlation.
Number of unnecessary antibiotics	Log of patient visits for presumed viral condition		
Number of broad-spectrum antibiotics	Log of total antibiotic prescriptions		
Number of prolonged antibiotics	Log of total antibiotic prescriptions		

#### PHO Trial

The primary analysis will use Poisson regression and will follow the intention to treat principle. The unit of analysis will be the physician (Table 2). The model will be estimated using quasi-likelihood estimation with a scale parameter to account for clustering by physician. The first, more pragmatic analytic model will compare all intervention conditions grouped together against usual care. A second, exploratory model will focus only on intervention conditions and will include terms indicating the presence (+) or absence (−) of each of the factors (intervention components) using effects coding [28]. The effect of each intervention component will be expressed as relative risk (RR) with 95% confidence intervals. Pairwise comparisons between physicians will be obtained from the model. To examine long-term effects of the intervention, we will repeat the analyses at 12 months post-randomization.

### Subgroup analysis

In both trials, we will explore differences in effects by physician characteristics (sex, years in practice, patient practice volume, continuity of care score, proportion of emergency room practice, proportion nursing home practice, practice complexity score (SAMI), proportion of practice > 85 years, rurality of practice address, neighbourhood income quintile of practice address and baseline prescribing rates). We will also look at the effects of intervention on prescriptions that were filled on the same day as a virtual visit.

### Sample size

#### PHO Trial

A total sample size of 3,136,000 patient visits over 6 months, obtained via an expected 6000 physicians meeting eligibility criteria (approximately 1200 usual care and 4800 intervention; 2400 with any factor present and 2400 with that factor absent) with an estimated average of 784 patient visits per cluster, achieves 83% power to detect a 7.5% relative reduction in antibiotic prescribing rate comparing the intervention to usual care, using a two-sided test for the difference between two Poisson rates. The between-cluster coefficient of variation was assumed to be 75%.

#### OH Trial

A total sample size of 3,136,000 patient visits over 6 months, obtained via an expected 4000 physicians within approximately 1000 offices meeting eligibility criteria (approximately 2000 physicians and 500 offices to each arm) with an estimated average of 784 patient visits per physician cluster and about 3136 patient visits per office cluster, achieves 80% power to detect a 13% relative reduction comparing the intervention to usual care. This would equate to a difference from 0.093 (93 per 1000 visits) to 0.081 (or 81 per 1000 visits), a relative risk of 0.87, using a two-sided test for the difference between two Poisson rates. The between-cluster coefficient of variation was assumed to be 75%.

### Process evaluation

The mixed-methods process evaluation may reveal why or why not the intervention worked and how individual factors can affect physician motivation, willingness, and ability to engage in new practices [29]. This theory-based process evaluation seeks to assess the perceived impact and underlying mechanism(s) of action of the components of the antibiotic prescribing A&F intervention. We will conduct three sets of data collection: theory-informed questionnaire; brief telephone calls to assess fidelity; and in-depth, theory-informed telephone interviews.

### Methods—process evaluation

#### Intervention fidelity

The relative effectiveness of intervention design-components can only be assessed if the recipients of the feedback receive and read the reports. Intervention fidelity will be measured according to the fidelity framework developed by Bellg and colleagues [30, 31]. We will assess if the A&F was *delivered*, *received*, and *acted on*.

#### PHO Trial

To assess if the intervention was *delivered* and *received*, the study team will check whether physicians have reviewed the antibiotic letter. At 7 months post intervention delivery, a research team member at PHO will contact a stratified random sample of participants from the PHO Trial by phone. Specifically, they will sort physicians by 10-year age group, sex, and area code, and randomly sample from each stratum until 300 physicians have been called. Each physician selected will be called up to 3 times over the course of 1 week. Non-respondents to phone calls will be tracked. Those physicians who answer the call will be asked whether they received the letter from PHO (Y/N), if they reviewed the letter (Y/N), and if they can confirm what the main message was. A sample of 300 physicians was calculated based on a total population of 4000 physicians that will be sent the intervention, a 30% opening rate based on our previous trial of sending antibiotic letters and Canadian data on letter opening rates [32], and a margin of error of 5%.

#### OH Trial

OH will track email read-receipts/open-rates for those receiving their *MyPractice*: Primary Care report.

#### PHO and OH Trials

Via surveys and in-depth interviews described below, we will measure if the intervention was *enacted* by asking physicians if they have used various components of the letter (e.g. viral prescription pad, communication tips, recommendations for shorter durations).

### Questionnaire to examine mechanism of action

The aim of the quantitative component of the process evaluation is to explore the effects of the interventions on the theory-based constructs hypothesized to explain behaviour change resulting from the intervention. We will use questionnaires and the AACTT framework (Action, Actor, Context, Target, Time) to specify the targeted behaviour [33]:

Action: prescribing antibiotics

Actor: primary care physician

Context: community-based clinic

Target: patients presenting to a community physician with infection-like symptoms

Time: any patient-clinician interaction for a condition where an antibiotic is considered

### Theoretical model: Health Action Process Approach

**Fig. 6:**
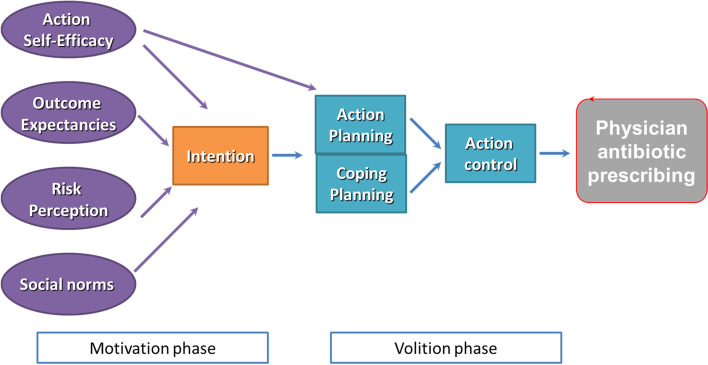
Proposed mechanisms of action, informed by the health action process approach [34, 35]

The process evaluation design was informed by the Health Action Process Approach (HAPA; Fig. 6) [34, 35]. HAPA is a behaviour change theory used to describe, explain, and predict behaviour and is useful to inform which strategies to include in interventions. HAPA postulates that a two-phase process influences behaviour, with each phase requiring different intervention strategies: a motivation phase where an individual forms an intention to engage in the behaviour and a volition phase involving translating that intention into actual behaviour. The motivation phase is influenced by (i) participants’ perceptions of the risk of adverse events related to engaging in the behaviour (*risk perception*); (ii) what they expect the outcomes of enacting the behaviour might be (*outcome expectancies*); and (iii) the belief in their capability to perform the desired action (*self-efficacy*). We also included a “social norms” construct (i.e. perceptions about what colleagues expect) as behaviour change theories suggest that feedback may work via this construct [36]. The volitional phase is broken down into the individual’s development of plans specifying when, where, and how they will enact the behaviour (*action planning*); plans for how they will overcome specific anticipated barriers they may face (*coping planning*); and the processes by which an individual self-monitors, is aware of the standards against which they are being compared, and whether they have made effort to address the discrepancy between what they are doing and the (*action control*). We will evaluate the hypothesized direct and indirect effects of the antibiotic letter on the HAPA constructs. We will map the factors (see Fig. 6) tested in the letter to the HAPA theory to assess how the intervention impacted intention in the motivation phase (via action self-efficacy, outcome expectancies, risk perception, descriptive norms, intention), and physician prescribing behaviours in the volitional phase (via action planning, coping planning, action control).

### Recruitment

#### OH Trial

Physicians (intervention and control) will receive an invitation to complete the process evaluation survey 1 month after receiving their *MyPractice*: Primary Care report. Since the delivery of *MyPractice*: Primary Care reports is via email, we will offer only the online survey platform. Physicians will be instructed that completion of the survey implies consent. Up to three email reminders to complete the survey will be sent.

#### PHO Trial

One month after receiving the first intervention letter, intervention participants (*N* ≈ 3200) will receive a second copy of their intervention letter, as well as an invitation to complete a survey. Physicians will be informed that completion of the survey implies consent. The completed survey can be returned via mail with a stamped return envelope, faxed, or through an online survey link. For both trials, the online survey will be administered using Qualtrics©, a secure online survey platform (https://www.qualtrics.com/). We will provide $20 (CDN) in the form of an electronic gift card, in recognition of the time required to complete the survey. The survey links will be disabled after the required number of survey responses is collected to preserve budget (see “Sample size” below). Participants who return paper copies of the survey will be reimbursed regardless.

### Data collection

The questionnaire will include demographic questions plus validated survey questions (with adaptations when necessary) to address relevant constructs (e.g. risk perception, outcome expectancy) related to intention and behaviour (e.g. action planning, coping planning) as defined by the HAPA [34]. The questionnaire will also include questions on *acceptability* as defined by the Theoretical Framework of Acceptability [37]. The framework defines acceptability as a multi-faceted construct that reflects the extent to which people delivering or receiving a healthcare intervention consider it to be appropriate, based on anticipated or experienced cognitive and emotional responses to the intervention. The questionnaire will assess the theoretical framework of acceptability (TFA) seven component constructs: affective attitude, burden, perceived effectiveness, ethicality, intervention coherence, opportunity costs, and self-efficacy [30]. Finally, the questionnaire will include questions on self-report of antibiotic prescribing relating to examples in the letter: (1) sinusitis, (2) pharyngitis, (3) bronchitis.

### Analysis of data from questionnaire

#### Sample size

The survey will be distributed to all physicians randomized to receive A&F in the PHO Trial (estimated *N* = 3200) and all physicians receiving the *MyPractice*: Primary Care reports in the OH Trial (estimated *N* = 4000). We anticipate a 20% response rate, based on previous mail surveys of physicians in Canada [32]. A sample size of 400 surveys from each trial (800 total) is deemed sufficient based on previous studies using this methodology [38]. A sample size of 400 survey respondents per trial achieves > 80% power to detect a small standardized regression coefficient of < 0.1 in a linear regression analysis at a 2-sided 5% significance level.

### Data analysis

Internal consistency will be assessed of theoretical constructs using Omega Squared [39]. If internal consistency is < 0.7, we will explore whether we will omit individual items. We will then calculate the mean of the items measuring each construct to create a summary score ranging from one to five. We will calculate means and standard deviations (SD) of all measured constructs, computed separately for the six experimental factors.

We will use multiple mediation regression models to explore whether the antibiotic letter worked through hypothesized pathways using the Hayes PROCESS mediation macro [40, 41]. Our aim is to assess the effects of three components of the A&F intervention, corresponding to research question 2: (i) adjusted comparator, (ii) emphasis on antibiotic harms, and (iii) viral prescription pad (all recipients of A&F in the PHO Trial receive a link to the viral prescription pad as part of the intervention). We will build two models: Model A: indirect effects of the letter component on intention via motivational constructs and Model B: indirect effects of the letter on antibiotic prescribing behaviour via planning and control constructs.

**Fig. 7:**
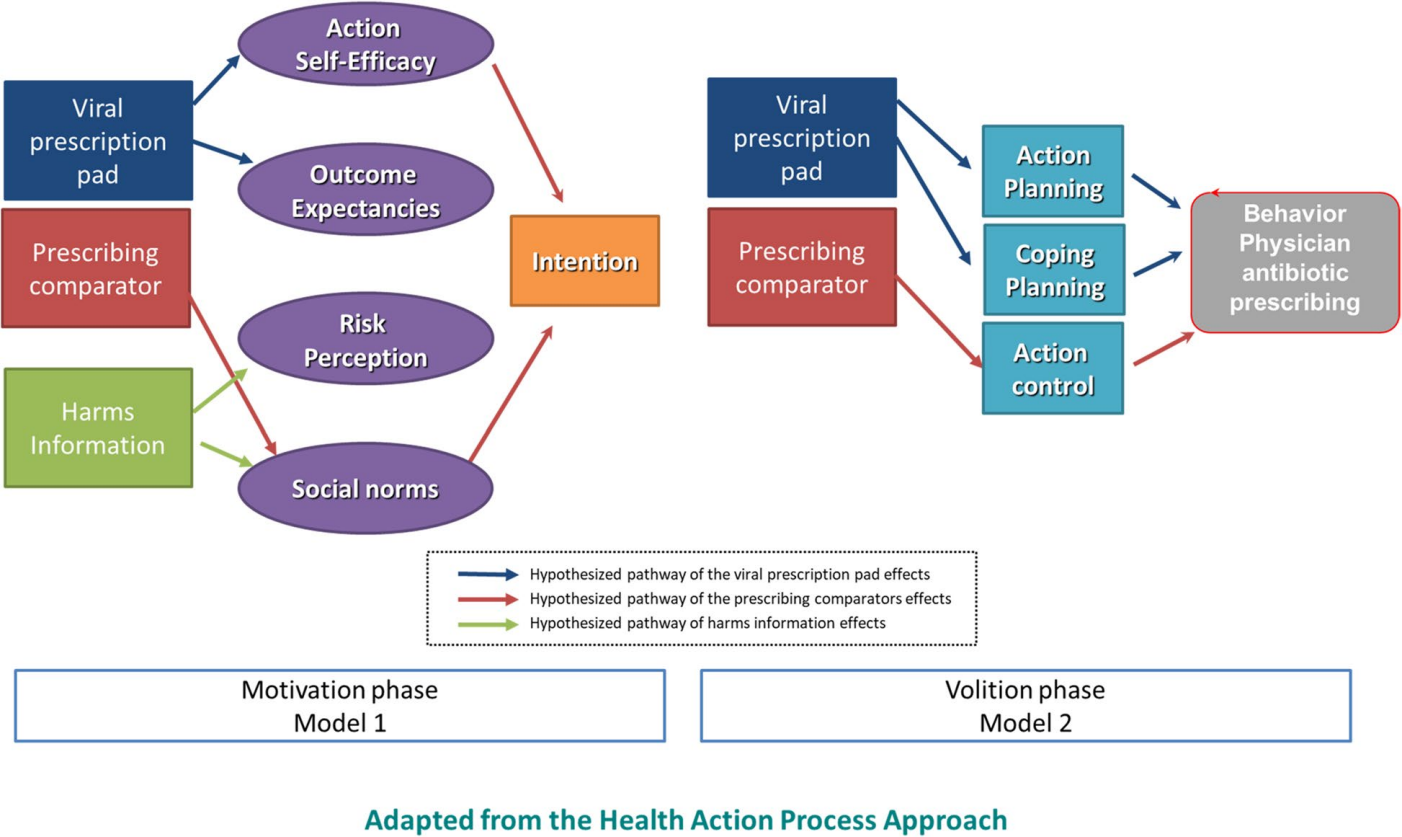
Multiple mediation regression models examining the effect of viral prescription pad, prescribing comparator, and harms information [34]

The theoretical models outlining the relationships between hypothesized predictor constructs, and the behaviour are summarized in Fig. 7. Such a model allows the direct and/or indirect effects of an independent variable X on a dependent variable Y through one or more mediators (M) to be estimated. The model will allow us to estimate the mechanism pathway through which intervention components (X) exert their effect on intention and prescribing behaviour (Y) is dependent on the value of the mediators (HAPA constructs). Each model will be tested using the appropriate subsample of trial participants. For the PHO Trial, we will assess the effects of intervention components (i.e. adjusted comparator, and harms information). In the OH Trial, we will assess the effects of the mailed viral prescription pad/emphasis on the prescription pad. The macro allows multiple mediator variables to operate in parallel and for the conduct of Poisson Regression to assess the impact on continuous outcomes (intention and prescription behaviour). Separate analyses will be conducted for each of the three intervention components.

### Qualitative component of the process evaluation

A qualitative approach was used to gain a nuanced understanding of how and why the intervention worked (or did not work) as intended [42]. We will use the Clinical Performance Feedback Intervention Theory (CP-FIT) to guide the development of the interview guide and the analysis [43]. The theory is based on a synthesis of qualitative research on feedback interventions. CP-FIT states that effective feedback works in a cycle of sequential processes. We will purposefully explore this process: goal setting, data collection and analysis, feedback, recipient Interaction, perception, and acceptance of the feedback, followed by intention, behaviour, and clinical performance improvement. We will also aim to understand which process of the CP-FIT the recipient is at, and what enabled them or prevented them from progressing through the different stages.

### Data collection

We will aim to recruit 4–8 physicians from each of the following groups: (i) PHO Trial, adjusted comparator, (ii) PHO Trial, unadjusted comparator, (iii) PHO Trial, harms emphasis, (iv) PHO Trial, no harms emphasis, and (v) OH Trial, mailed viral prescription pad/emphasis. Participants will be purposively sampled to allow for variation in age, gender, experience, and clinical context. Participants will be screened to confirm they have read the letter.

#### Recruitment strategies

At the end of the process evaluation survey, physicians will be invited to participate in the qualitative interview.

If required, we will randomly select physicians based on our purposive sampling strategy. These physicians will be sent a letter inviting them to participate.

If required, snowball sampling will be used by asking physicians if they know of others who received the letter and would be willing to speak about it.

If required, we will use social media (Twitter, LinkedIn, etc.) to invite participants.

The study team will reach out to physicians interested in participating in an interview. They will be provided with an information letter and consent form. Physicians who complete the interview will be provided an honorarium of $100 in the form of an electronic gift card.

### Interview guide

Our study objectives and interview questions are guided by the Brehaut et al. [44] suggestions for optimizing A&F effectiveness, and the Clinical Performance Feedback Intervention Theory (CP-FIT) [43]. We will explore which stage of the CP-FIT the physician is at (e.g. perception, acceptance, intension, and behaviour) and what enabled them to get to that point and prevent them from moving to the next process.

The interview guide will also aim to explore HAPA constructs [34, 35]: *risk perception, outcome expectancies, self-efficacy, action planning, coping planning and action control*. We will explore the underlying mechanisms through which the intervention may or may not have influenced antibiotic prescribing intention, action planning, and antibiotic prescribing behaviours, particularly related to initiation and duration of a prescription. The semi-structured guide also includes questions assessing intervention fidelity according to the fidelity framework developed by Bellg and colleagues [30, 31].

Interviews will last approximately 45 min, conducted by Zoom and recorded for transcription, coding, and analysis. Brief demographic questions will be asked at the beginning of the interview, including type of letter received, physician gender, years in practice, type of practice, location of practice, and average number of patients seen a day.

### Analysis

Data analysis will occur iteratively using NVIVO 12. Thematic analysis will consist of six stages, informed by the approach of Braun and Clarke [45]: (1) familiarization of the interview in its entirety; (2) the production of initial deductive and inductive codes from the data will be performed by multiple coders and we will explore (CP-FIT) variables and explanatory mechanisms while allowing for new codes on emerging issues; (3) we will review generated codes among coders to ensure general consensus across coders; (4) searching for themes, we will begin to explore the relationship between codes, between themes and how they relate to the intervention content; (5) revising and summarizing themes using thematic mapping to explore relationships between themes, will involve discussions with multiple team members representing multiple disciplines; and (6) writing the report. Sample size for interviewing will be informed by saturation from participants from each of the study arms [46]. Qualitative data will be analysed iteratively so that themes that emerge in early interviews can be explored in later ones.

### Data synthesis and triangulation

Quantitative and qualitative data will be compared and contrasted to explore consistencies and contradictions across the dataset using the methods described by Hopf et al. [47]. Comparison between HAPA constructs and qualitative data will allow us to use the data from interviews to explain quantitative findings. We will triangulate data that explore key questions regarding the embedded process evaluation, including mechanism of action and effects of the intervention. For example, regarding mechanism of action, we will compare and integrate findings from participant interviews and quantitative data regarding self-efficacy, risk perception, outcome expectancies, and intentions of changing behaviours in antibiotic prescribing. A synthesis of findings from different sources will be used to highlight key mechanisms, implementation difficulties, and outcomes of the study [48, 49].

## Economic evaluation

If the A&F interventions are shown to be effective, we will conduct a cost-effectiveness analysis of the intervention compared to usual care from a perspective of Ontario Health. Total costs will include the cost of the intervention and health system costs. We will track the time and costs required to deliver the intervention, including time for intervention development and quality assurance/monitoring. Health system costs will be derived using existing cost macros available at ICES [50]. Consistent with the trial, effectiveness will be measured as antibiotic prescribing rate. Analyses will conform to the most recent Canadian guidelines for economic evaluation [51] and current guidelines for such analyses alongside randomized control trials [52]. The incremental cost and outcome will be estimated using generalized linear models with appropriate link functions and distributions. We will evaluate uncertainty of the cost-effectiveness estimates using non-parametric bootstrapping. Results from the bootstrapping exercise will also be used to estimate 95% confidence intervals and depict cost-effectiveness acceptability curves, which show the probability of A&F interventions being cost-effective to a range of potential threshold values that the health system may be willing to pay for an additional unit of effect [53].

## Patient and public involvement

We involved members of the public to solicit input about the intervention content. Our lead patient partner helped to gather input from additional patients and collaborated with the design team to iteratively refine the intervention materials. This was especially important for the section of the intervention letter which provided advice to family physicians about what to say to patients who seemed to be requesting antibiotics. Since the intervention is directed to family physicians, we also sought input on the intervention materials from relevant organizations including Choosing Wisely Canada, the Ontario College of Family Physicians and Ontario Health, who each have their own mechanisms for soliciting patient and public input on their strategies and initiatives. We plan a lay summary of the results and implications, which will be disseminated through partnerships with relevant organizations.

## Discussion

Collectively, these two trials involve provision of antibiotic A&F to nearly all family physicians in Ontario, Canada. The work pursues the dual aim of improving quality of care and advancing scientific understanding of how to optimize A&F. A key strength of this project is the stakeholder partnerships, which enable conduct of this work at scale in our implementation science laboratory [14]. We acknowledge several limitations as well. First, the OH Trial has no control arm without A&F; usual care for participants in the OH Trial is to receive A&F and OH was unable to implement an antibiotic module in their *MyPractice*: Primary Care report for only a subset. Fortunately, we were able to retain a control arm in the PHO Trial. Second, due to data limitations, the feedback in both trials includes antibiotic prescribing only for those over age 65 and cannot be used to assess clinical appropriateness. Our prior work has shown that antibiotic prescribing for those over age 65 is a good proxy for antibiotic prescribing in ages by these clinicians (Additional File 2) and we know from previous trials that a large proportion of antibiotics prescribed in this age group are clinically inappropriate [23]. We anticipate this issue will be explored further in the qualitative aspect of the process evaluations. Third, the trials will occur while the COVID-19 pandemic continues, where there is a high level of burnout and antibiotic prescriptions patterns and primary care has changed [12]. Again, the qualitative aspect of the process evaluation may shed light on this issue. Fourth, prolonged antibiotic duration was measured as more than 7 days because most outpatient-treated infections should be treated with 7 or fewer days of antibiotics; however, there are circumstances where a longer duration is required. Fifth, in the OH Trial, there is some risk of contamination with the virtual prescription pad as it is possible some offices that shared EMRs were not captured in the cluster randomization. Sixth, regarding recruitment for the process evaluation, we recognize that those who answer the survey and accept an invitation for an interview will likely differ from those that do not. Regarding the survey, we will compare characteristics between those that respond and those that do not, and we will interpret results accordingly. For the interviews, we will aim to recruit from a diverse sample. Finally, Ontarian administrative data does not capture race and ethnicity data and therefore we cannot explore the effects that these characteristics might have on our outcomes; however, we will explore these questions in the process evaluation. We anticipate that the findings from this research program will inform ongoing policy and practice for our stakeholders and partners about community-based antimicrobial stewardship programs.

### Group authorship statement

The following authors are part of the Ontario Healthcare Implementation Laboratory study team: Monica Taljaard, Jeremy M. Grimshaw, Meagan Lacroix, Mina Tadrous Valerie Leung, Kevin Brown, Andrew M. Morris, Gary Garber, Justin Presseau, Kednapa Thavorn, Jerome A. Leis, Holly O. Witteman, Jamie Brehaut, Nick Daneman, Michael Silverman, Michelle Greiver, Tara Gomes, Michael R. Kidd, Jillian J. Francis,Merrick Zwarenstein, Jonathan Lam, Cara Mulhall, Sharon Gushue, Sukhleen Uppal, and Andrew Wong.

## Trial status

The two trials will launch in January 2021.

## Supplementary Information

**Additional file 1:** CONSORT 2010 checklist of information to include when reporting a randomized trial

**Additional file 2:** Correlation of antibiotic prescribing between 65+ with for all ages in primary care

**Additional file 3:** The Pragmatic-explanatory continuum indicator summary 2 provider strategies (PRECIS-2-PS) wheel.

**Additional file 4:** Cover Letter to Accompany Mailed Viral Prescription Pad

**Additional file 5:** Example Physician Letter for Public Health Ontario Trial

**Additional file 6:** Definition of unnecessary antibiotic prescription

## Data Availability

Data sharing is not applicable to this article as no datasets have been generated yet.

## Abbreviations

AACTT: Action Actor Context Target Time
AF: Audit and feedback
APR: Antibiotic prescribing rate
AVI: Antibiotics prescribed for viral infections
BCT: Behaviour Change Technique
CAD: Coronary artery disease
CAPE: Client Agency Program Enrollment
CHF: Congestive health failure
CI: Confidence interval
CIHI-DAD: Canadian Institute for Health Information – Discharge Abstract Database
CKD: Chronic kidney disease
CWC: Choosing Wisely Canada
COPD: Chronic obstructive pulmonary disease
CP-FIT: Clinical Performance Feedback Intervention
CPSO: College of Physicians and Surgeons of Ontario
DIN: Drug Identification Number
DOT: Days of therapy (antibiotics)
EMR: Electronic Medical Record
HAPA: Health Action Process Approach
ICD: International Classification of Diseases
ICES: Institute for Clinical Evaluative Sciences
IPDB: ICES Physician Database
MOST: Multiphase Optimization STrategy
OCR: Ontario Cancer Registry
ODB: Ontario Drug Benefit
ODD: Ontario Diabetes Dataset
OH: Ontario Health
OHIP: Ontario Health Insurance Program
OMID: Ontario Myocardial Infarction Dataset
PHO: Public Health Ontario
REB: Research Ethics Board
RPDB: Registered Persons Database
RR: Relative risk
SAMI: Standardized ACG Morbidity Index
SAS: Statistical Analysis System
SD: Standard deviation
TFA: Theoretical framework of acceptability
TCPS2: Tri-Council Policy Statement
WCH: Women’s College Hospital
