# Supplementary material for: Optimizing responsiveness to feedback about antibiotic prescribing in primary care: protocol for two interrelated randomized implementation trials with embedded process evaluations
Source: Implement Sci. 2022 Feb 14;17:17. doi: 10.1186/s13012-022-01194-8 (PMC8842929; doi:10.1186/s13012-022-01194-8)
Supplement: Supplementary file 2 — Additional file 2: Correlation of antibiotic prescribing between 65+ with for all ages in primary care [file 13012_2022_1194_MOESM2_ESM.docx]

**Additional File 2**

Correlation of antibiotic prescribing between 65+ with for all ages in primary care

Administrative data from ICES contains medications prescribed through the ODB database for all seniors 65 years of age and older. Since we are unable to provide all antibiotic prescribing feedback to physicians, we sought to evaluate the correlation among Ontario family physicians between total antibiotic prescribing and antibiotics prescribed to those 65 years of age and older only. Using data from IQVIA Xponent database from January 1 to December 31 2019, we plotted the antibiotic prescribing rate (antibiotic prescriptions per 100 total prescriptions) and calculated Spearman correlation coefficients. We performed this analysis separately for male and female patients. Antibiotic prescribing by family physicians to patients 65 years of age and older is strongly correlated with total antibiotic prescribing (Spearman correlation of 0.80 and 0.84 for male and female patients, respectively).

Table S1. Descriptive Statistics and Spearman Correlation Between Total Antibiotic Prescribing and Antibiotics Prescribed to Males 65 Years of Age and Older

| Variable | N (physicians) | Mean (SD) | Median | Min | Max | **Spearman *r***  **Total Patient Population** | *p* |
| --- | --- | --- | --- | --- | --- | --- | --- |
| >65 year old male patients | 12683 | 9.35 (11.65) | 4.89 | 0.08 | 92.59 | **.80** | <0.0001 |
| Total patient population | 14238 | 12.16 (11.10) | 8.15 | 0.02 | 82.61 |  |  |


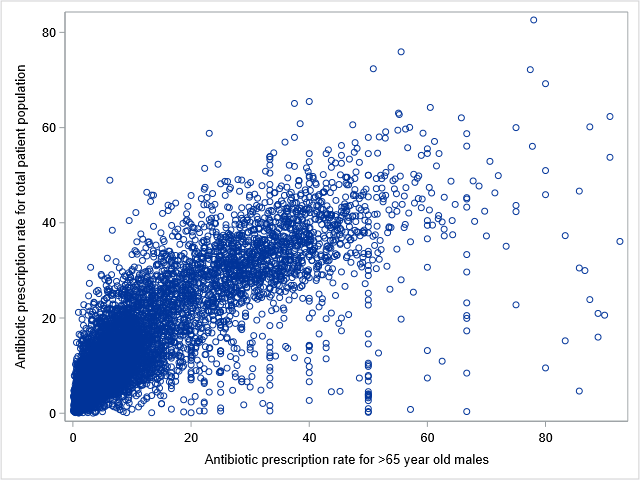


Figure S1: Comparing antibiotic prescription rate among >65 years old males to overall patient population

Table S2. Descriptive Statistics and Spearman Correlation Between Total Antibiotic Prescribing and Antibiotics Prescribed to Females 65 years of Age and Older

| Variable | N | Mean (SD) | Median | Min | Max | **Spearman *r***  **Total Patient Population** | *p* |
| --- | --- | --- | --- | --- | --- | --- | --- |
| >65 year old emale patients | 13103 | 10.83 (11.72) | 6.51 | 0.03 | 88.89 | **.84** | <.0001 |
| Total patient population | 14238 | 12.16 (11.10) | 8.15 | 0.02 | 82.61 |  |  |


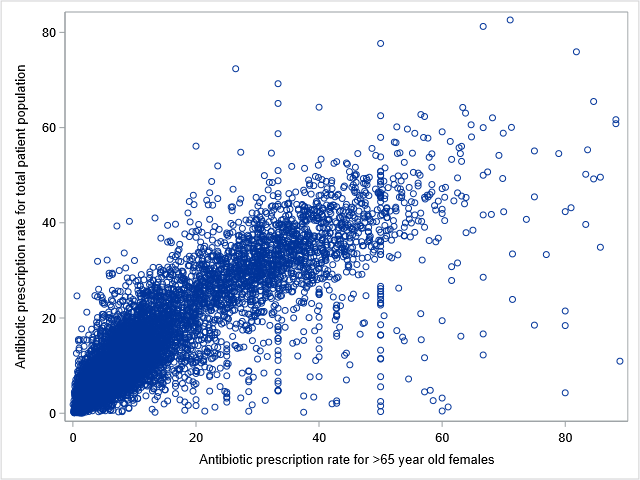


Figure S2: Comparing antibiotic prescription rate among >65 year old females to overall patient population
