# Supplementary material for: Optimizing responsiveness to feedback about antibiotic prescribing in primary care: protocol for two interrelated randomized implementation trials with embedded process evaluations
Source: Implement Sci. 2022 Feb 14;17:17. doi: 10.1186/s13012-022-01194-8 (PMC8842929; doi:10.1186/s13012-022-01194-8)
Supplement: Supplementary file 3 — Additional file 3: The Pragmatic-explanatory continuum indicator summary 2 provider strategies (PRECIS-2-PS) wheel. [file 13012_2022_1194_MOESM3_ESM.docx]

**Additional File 3**

**The Pragmatic-explanatory continuum indicator summary 2 provider strategies (PRECIS-2-PS) wheel.**

These interrelated studies are considered to be a pragmatic RCT for the following reasons: (1) they evaluate the effectiveness of a potentially sustainable A&F intervention being introduced under usual conditions, across a jurisdiction; (2) eligible family physicians are identified through the use of existing provincial registries and enrolled in this study through a waiver of consent; and (3) data collection utilizes routinely collected information by existing administrative databases and data will be analyzed using intent-to-treat analysis (Additional File 3 and Table S3). The intention of this trial is to send A&F interventions when most antibiotics are prescribed, which is during the winter months.

**Table S3: Domain, rationale and points of the pragmatic-explanatory continuum indicator summary 2 provider strategies (PRECIS-2-PS) wheel**

| **Domain** | **Question** | **Rationale** | **Points** | |
| --- | --- | --- | --- | --- |
|  |  |  | **Ontario Health (OH) Trial** | **Public Health Ontario**  **(PHO) Trial** |
| **Eligibility** | To what extent are the healthcare professionals in the trial similar to those in usual care? | Those in the OH trial may differ as these physicians who signed up to receive a multi-topic audit and feedback report. The physicians in the PHO trial include all remaining eligible physicians. | 4 | 5 |
| **Recruitment** | How much extra effort is made to recruit healthcare professionals into the trial compared to what is available to encourage their engagement in usual care settings? | Eligible physicians are identified using existing provincial registries and enrolled in this study through a waiver of consent. | 5 | 5 |
| **Setting** | How different is the health care or public health setting (e.g., hospital, clinic, health department) in which the trial is conducted compared to usual care settings? | The setting is the same as regular clinical care. | 5 | 5 |
| **Implementation resources** | How different are the resources needed to support the delivery of the provider-focused strategies from resources that are readily available in usual care? | The OH trial is part of a running program and therefore are not different from usual care. The PHO trial is a new initiative but would not require substantial resources to continue if found to be effective. | 5 | 4 |
| **Flexibility of provider-focused strategies** | How different is the flexibility in how provider-focused strategies are delivered in the trial and the flexibility in how provider-focused strategies are likely to be delivered in usual care? | The intervention would not be delivered differently if it was integrated into usual care. | 5 | 5 |
| **Flexibility of intervention** | How different is the flexibility in how the intervention is delivered by healthcare providers to patients and the flexibility in how the intervention would be delivered in usual care? | Physicians can use the intervention as they choose after they receive the intervention. | 5 | 5 |
| **Data collection** | How different is the frequency and intensity of measurement and data collection throughout the trial compared to what is considered routine in usual care? | Data is routinely collected and is part of standard administrative databases. Extra resources would be required to pull and analyze the data. | 4 | 4 |
| **Primary outcome** | To what extent is the trial’s primary outcome important to healthcare professionals? | Our primary outcome is of concern to public health professionals and those interested in antimicrobial stewardship but may not be considered top priority by primary care physicians. | 3 | 3 |
| **Primary analysis** | To what extent are all data included in the analysis of the primary outcome? | We will be using intent-to-treat analysis. Data is routinely collected and will be analyzed for all trial participants. | 5 | 5 |
| **Total** | |  | 41/45 | 41/45 |
