# Supplementary material for: Optimizing responsiveness to feedback about antibiotic prescribing in primary care: protocol for two interrelated randomized implementation trials with embedded process evaluations
Source: Implement Sci. 2022 Feb 14;17:17. doi: 10.1186/s13012-022-01194-8 (PMC8842929; doi:10.1186/s13012-022-01194-8)

**Additional File 5**

Example Physician Letter for Public Health Ontario Trial

Version: Adjusted Peer Comparator and Harms Information


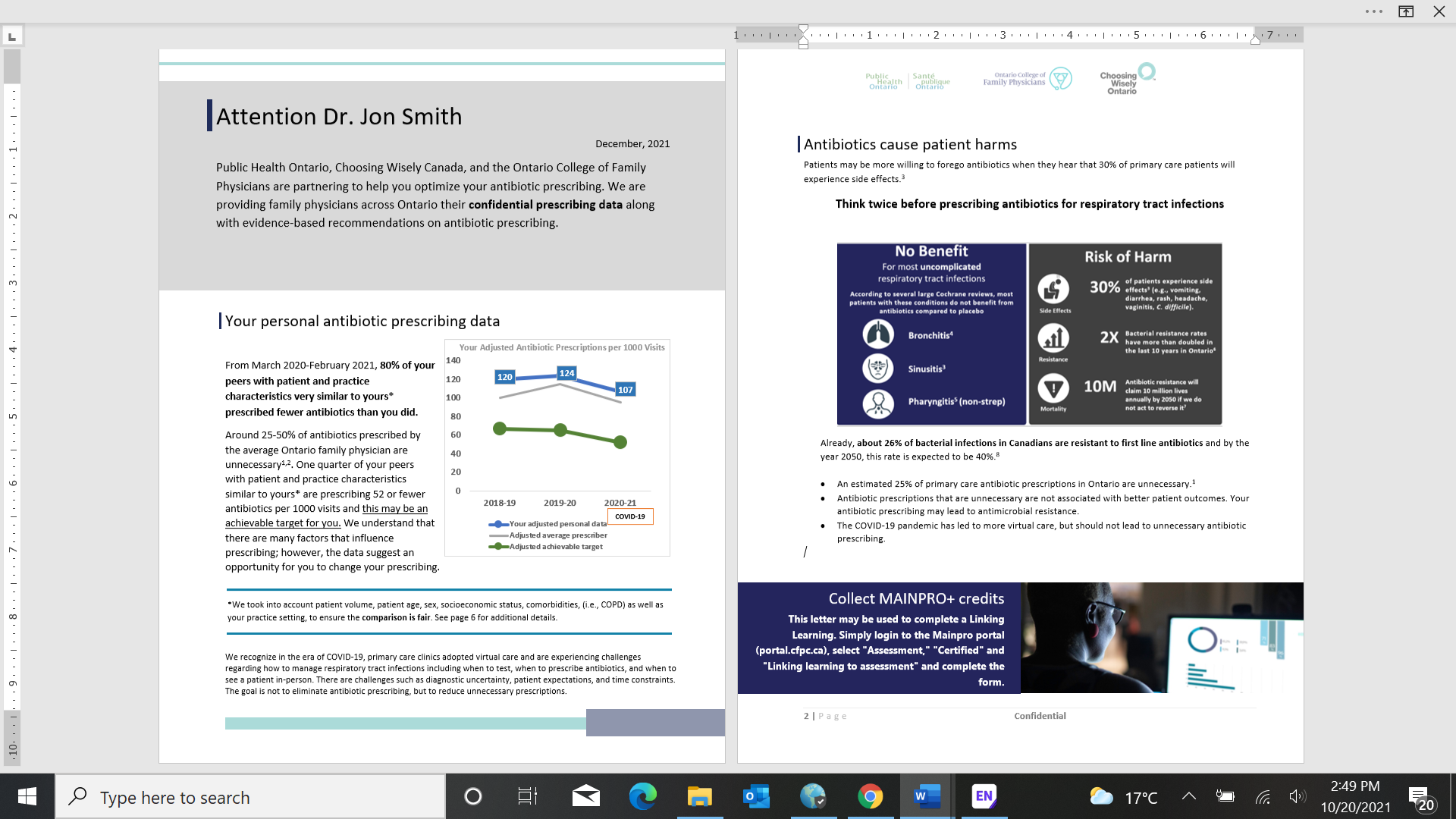


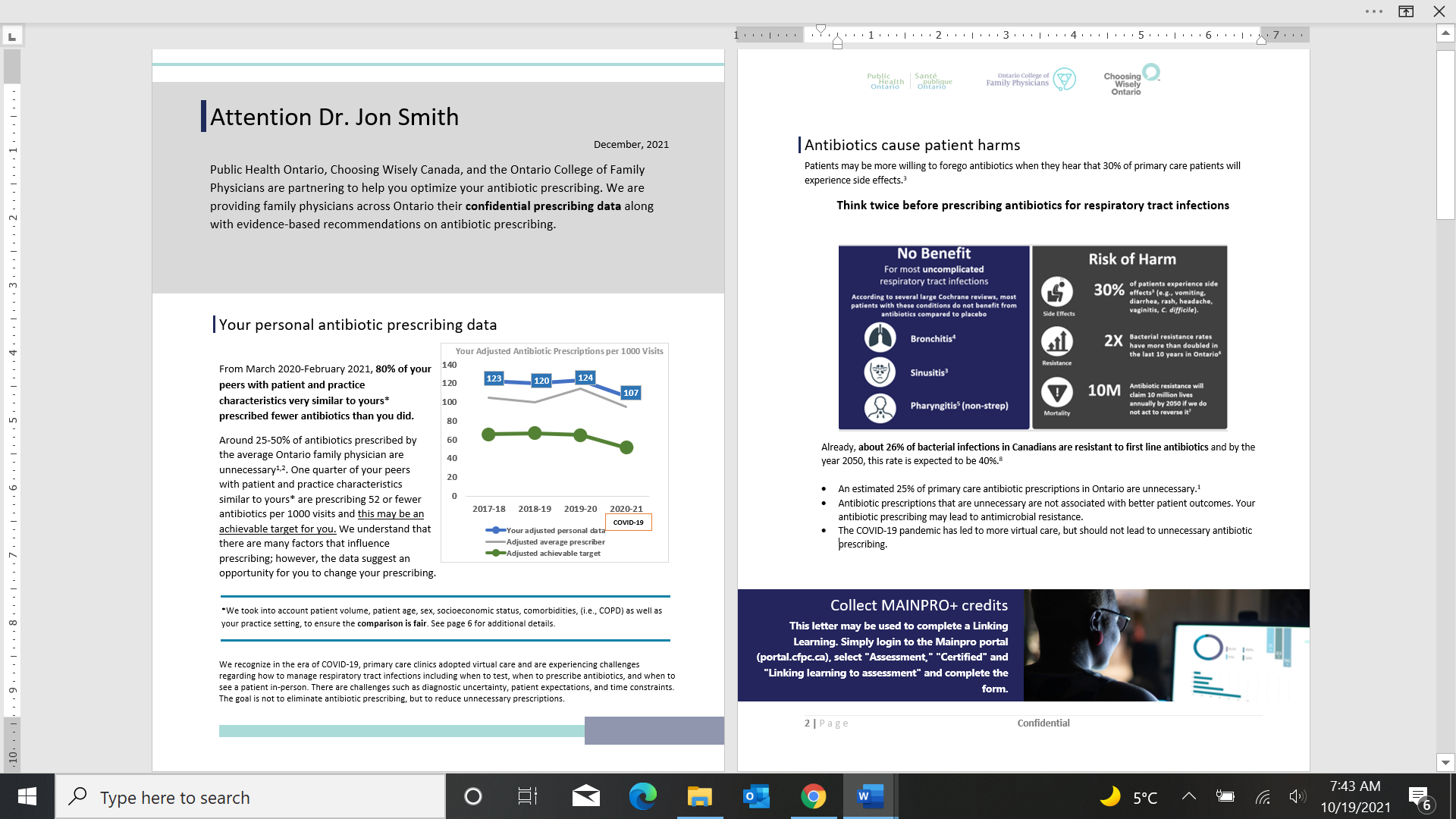


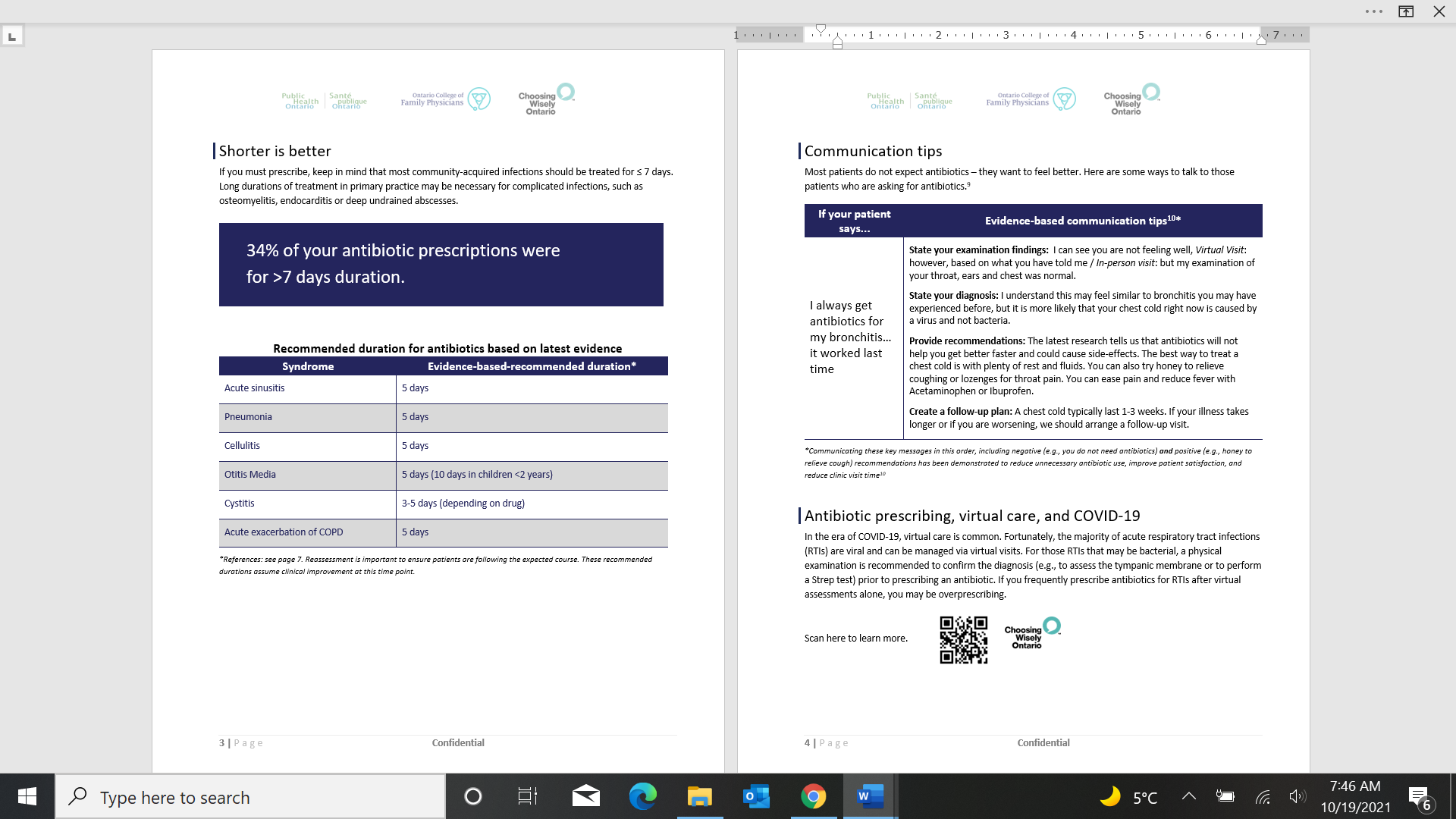


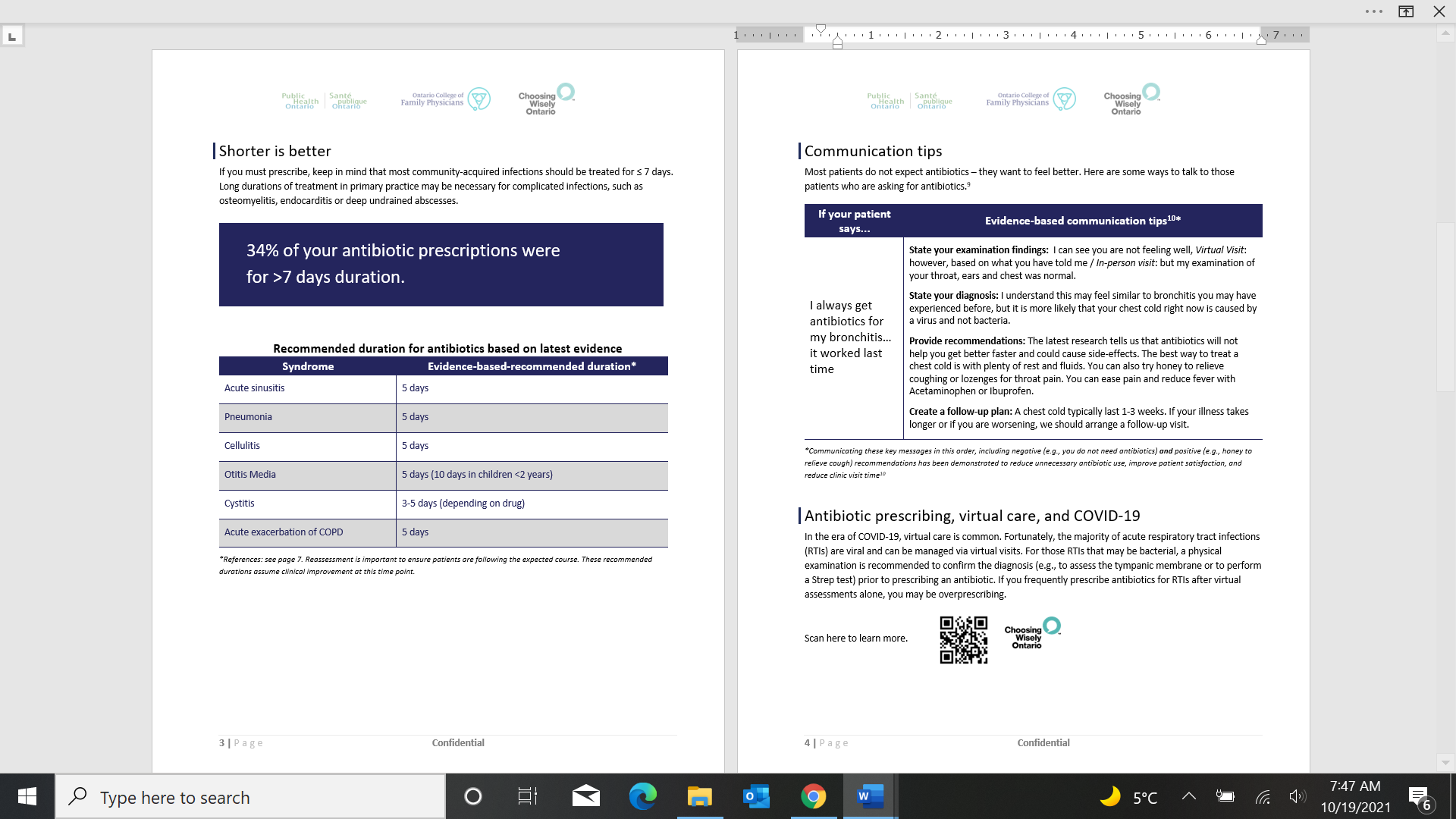


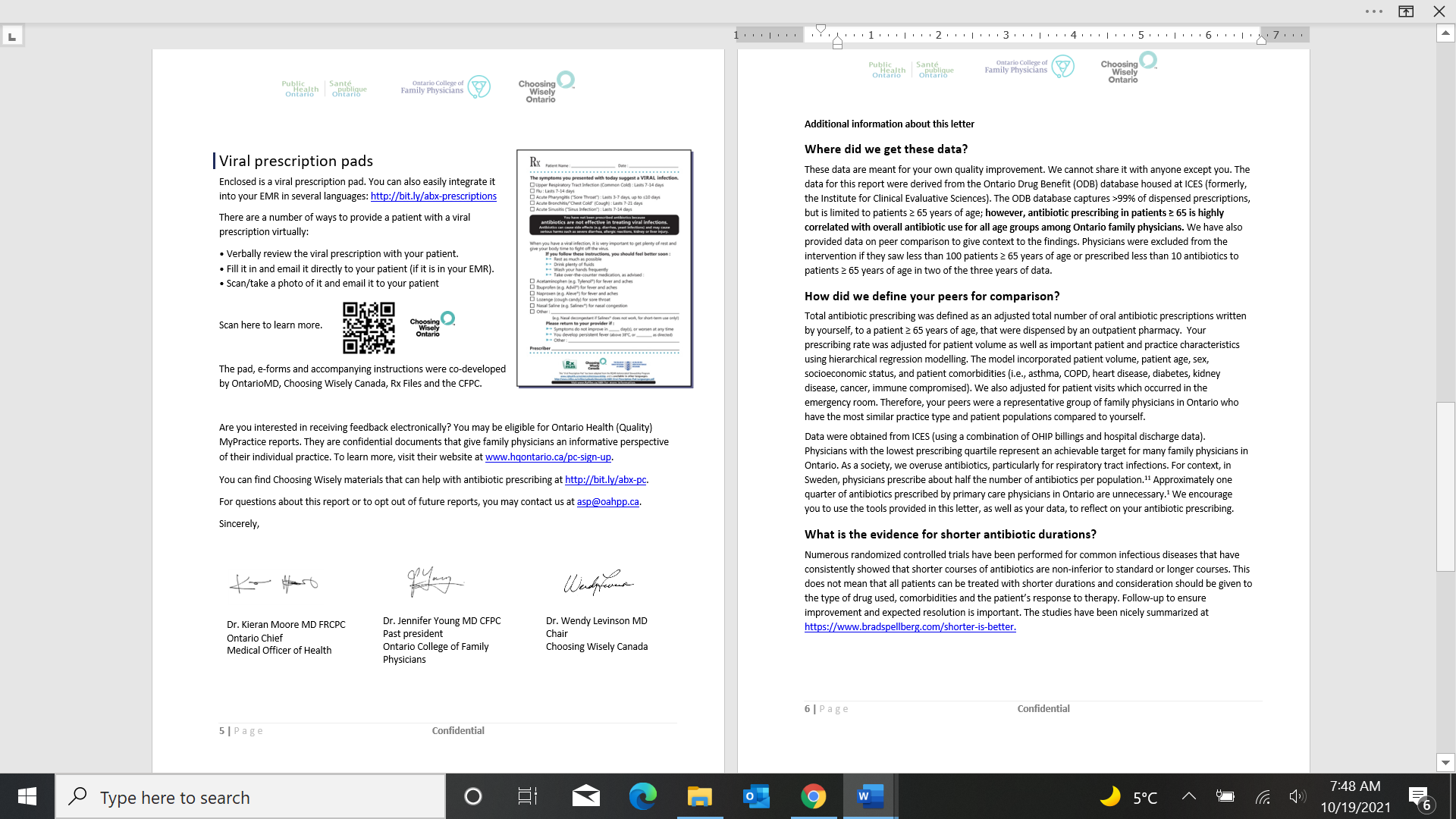


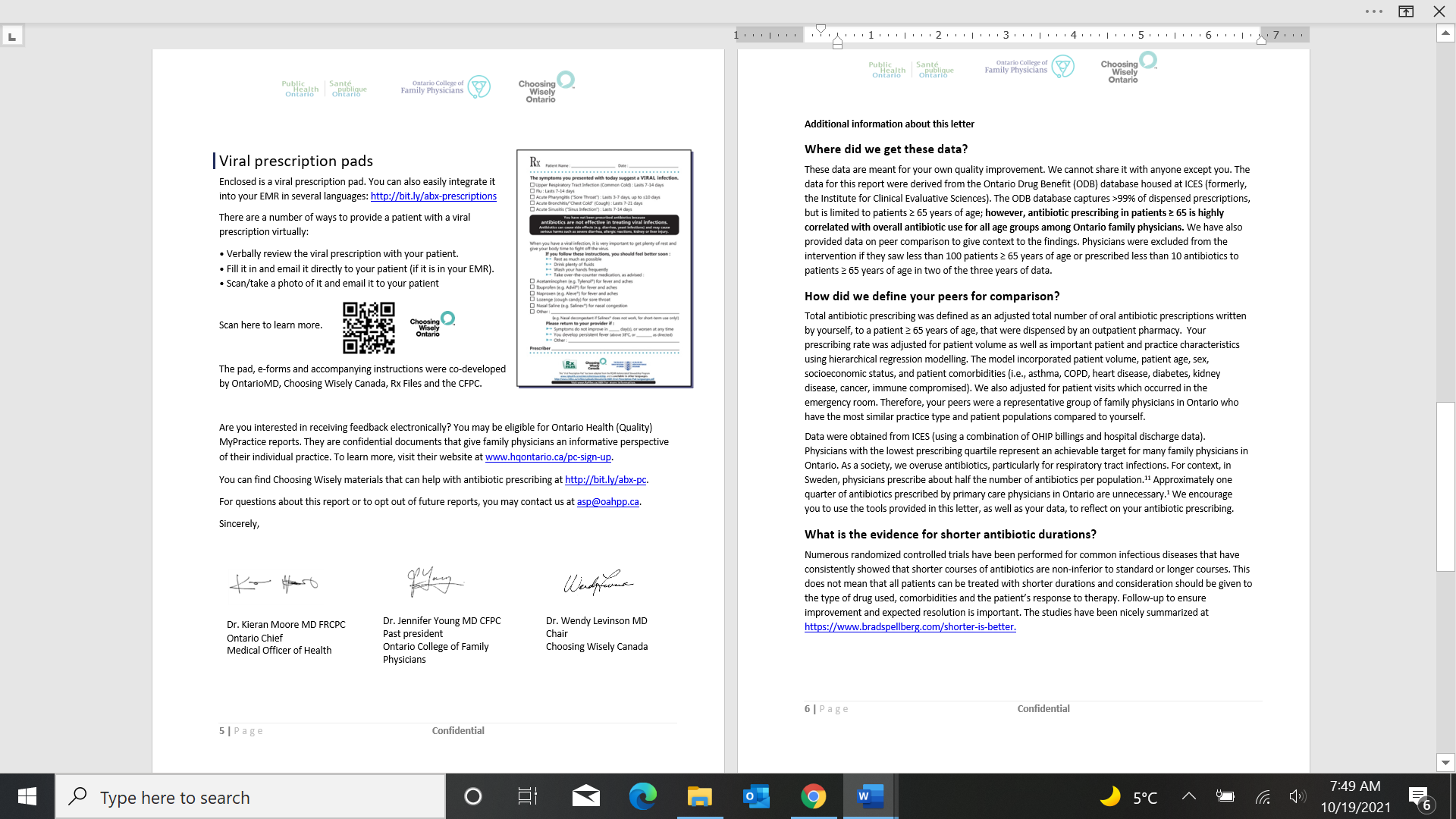


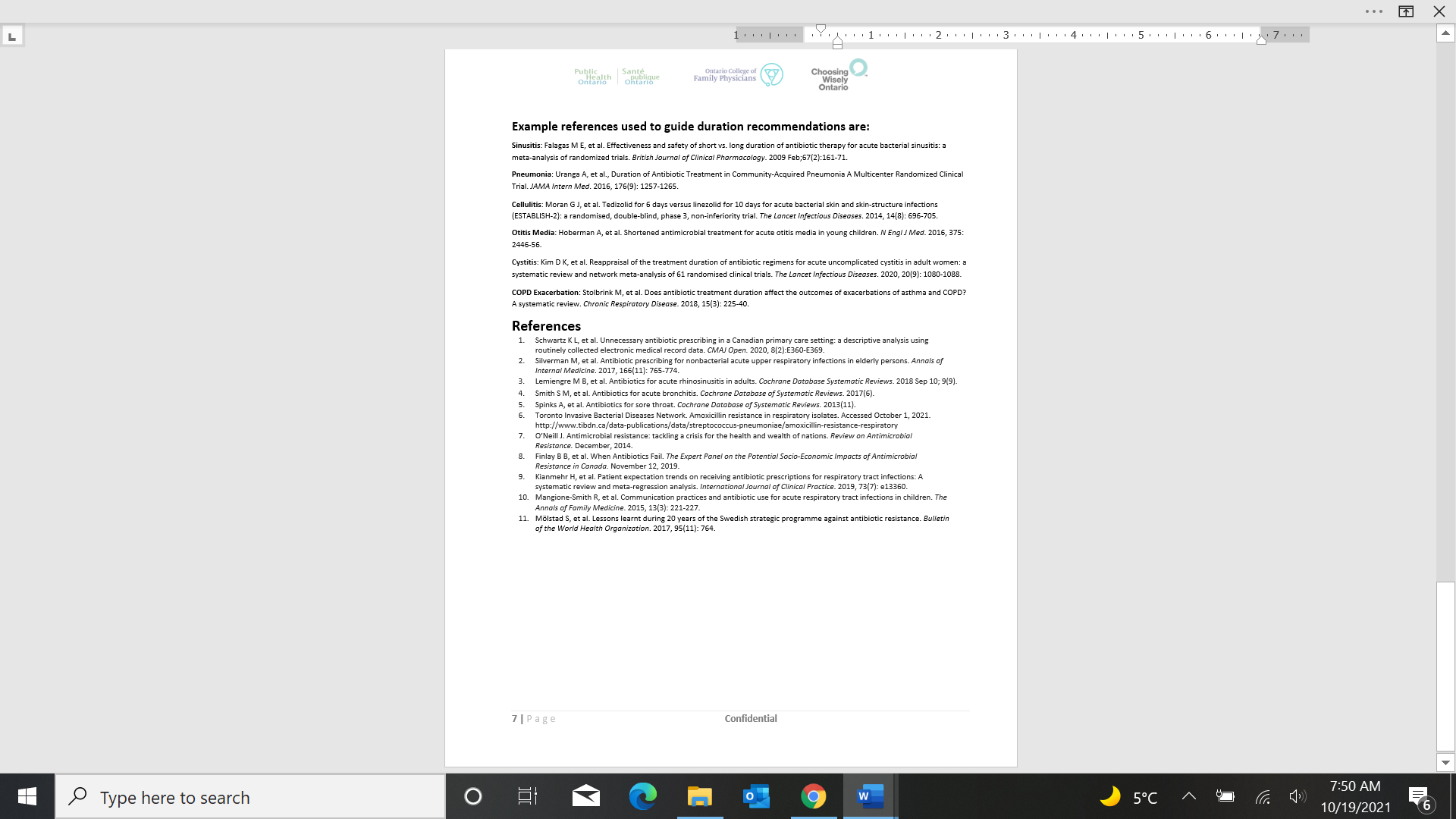

Supplement: Supplementary file 5 — Additional file 5: Example Physician Letter for Public Health Ontario Trial [file 13012_2022_1194_MOESM5_ESM.docx]
