# Supplementary material for: Optimizing responsiveness to feedback about antibiotic prescribing in primary care: protocol for two interrelated randomized implementation trials with embedded process evaluations
Source: Implement Sci. 2022 Feb 14;17:17. doi: 10.1186/s13012-022-01194-8 (PMC8842929; doi:10.1186/s13012-022-01194-8)
Supplement: Supplementary file 6 — Additional file 6: Definition of unnecessary antibiotic prescription [file 13012_2022_1194_MOESM6_ESM.docx]

**Additional File 6**

**Definition of unnecessary antibiotic prescription**

Definition: Number antibiotic prescriptions /100 visits for a viral respiratory condition (defined below)

Definitions, diagnostic tests, and OHIP codes to determine inclusion in outcome assessment of inappropriate antibiotic prescribing [55].

Exclusion Criteria: Any case with a test done (Table S1) 14 days before or 30 days after the Table S2 code OR if any bacterial diagnosis 14 days before to 30 days after (Table S3). Also exclude those with immune suppression, active cancer, or long-term care residents.

Table S4: Viral Respiratory Illness Definitions and OHIP Codes

| **Description** | **OHIP Code** |
| --- | --- |
| Asthma | 493 |
| Common cold | 460, 464 |
| Influenza | 487 |
| Acute sinusitis | 461 |
| Acute bronchitis | 466 |

Table S5: Selected OHIP Fee Codes for Tests Indicating Possible Bacterial Infection

| **Description** | **Type of Test** | **OHIP Code** |
| --- | --- | --- |
| Diagnostic radiology chest, single view | Chest radiography | X090 |
| Diagnostic radiology chest, 2 views | Chest radiography | X091 |
| Diagnostic radiology chest, 3 or more views | Chest radiography | X092 |
| Sputum – culture and smear | Sputum | L629 |
| Sputum per specimen for general and/or specified assessment | Sputum | L716 |
| Blood (including aerobic, anaerobic, subcultures, smears) per bottle | Blood culture | L624 |
| Culture – throat swab, for streptococcus screen only | Throat swab | L640 |
| Urine, pour plate, or Miles-Misra quantitative | Urine culture | L633 |
| Urine calibrated volume to include plate, turbidimetric, or photometric techniques | Urine culture | L634 |
| Urinalysis, routine | Urine culture | G009 |
| One or more parts of above without microscopy | Urine culture | G010 |

Table S6: Selected ICD-9 and ICD-10 Codes in the OHIP Database and DAD That Are Used to Indicate Possible Bacterial Infection

| **Description** | **OHIP Diagnosis Codes (ICD-9)** |
| --- | --- |
| Typhoid and paratyphoid fevers | 002 |
| Other salmonella infections | 003 |
| Whooping cough, pertussis | 033 |
| Septicemia, blood poisoning | 038 |
| Actinomycotic infections | 039 |
| Other bacterial diseases | 040 |
| Syphilis, all sites and stages | 097 |
| Gonococcal infections | 098 |
| Other types of leukemia | 208 |
| Hypogammaglobulinemia, agammaglobulinemia, other immunity disorders | 279 |
| Suppurative otitis media | 382 |
| Acute tonsillitis | 463 |
| Chronic sinusitis | 473 |
| Pneumonia – all types | 486 |
| Chronic bronchitis | 491 |
| Emphysema | 492 |
| Bronchiectasis | 494 |
| Other chronic obstructive pulmonary disease | 496 |
| Pleurisy with or without effusion | 511 |
| Acute appendicitis, with or without abscess or peritonitis | 540 |
| Cholecystitis, without gall stones | 575 |
| Acute or chronic pyelonephritis, pyelitis, abscess | 590 |
| Cystitis | 595 |
| Non-specific urethritis (not sexually transmitted) | 597 |
| Prostatitis | 601 |
| Orchitis, epididymitis | 604 |
| Boil, carbuncle, furunculosis | 680 |
| Cellulitis, abscess | 682 |
| Acute lymphadenitis | 683 |
| Pilonidal cyst or abscess | 685 |
| Osteomyelitis | 730 |
| Lacerations, open wounds – except limbs | 879 |
|  | **DAD (ICD-10)** |
| Pneumonia due to *Streptococcus pneumoniae* | J13 |
| Pneumonia due to *Haemophilus influenzae* | J14 |
| Bacterial pneumonia, not elsewhere classified | J15 |
| Pneumonia due to other infectious organisms, not elsewhere classified | J16 |
| Pneumonia in diseases classified elsewhere | J17 |
| Pneumonia, organism unspecified | J18 |
| Simple and mucopurulent chronic bronchitis | J41 |
| Unspecified chronic bronchitis | J42 |
| Emphysema | J43 |
| Other chronic obstructive pulmonary disease | J44 |
| Bronchiectasis | J47 |
| Abscess of lung and mediastinum | J85 |
| Pyothorax | J86 |
| Respiratory failure, not elsewhere classified | J96 |
