# Supplementary material for: Optimizing responsiveness to feedback about antibiotic prescribing in primary care: protocol for two interrelated randomized implementation trials with embedded process evaluations
Source: Implement Sci. 2022 Feb 14;17:17. doi: 10.1186/s13012-022-01194-8 (PMC8842929; doi:10.1186/s13012-022-01194-8)
Supplement: Supplementary file 4 — Additional file 4: Cover Letter to Accompany Mailed Viral Prescription Pad [file 13012_2022_1194_MOESM4_ESM.docx]

**Additional File 4**
